# Factors Contributing to the High Malignancy Level of Cholangiocarcinoma and Its Epidemiology: Literature Review and Data

**DOI:** 10.3390/biology14040351

**Published:** 2025-03-28

**Authors:** Xuan Li, Renchu Guan, Shuangquan Zhang

**Affiliations:** 1 Key Laboratory of Symbolic Computation and Knowledge Engineering of Ministry of Education, College of Computer Science and Technology, Jilin University, Changchun 130012, China; xuanli0518@gmail.com; 2 School of Cyber Science and Engineering, Nanjing University of Science and Technology, Nanjing 210094, China

**Keywords:** cholangiocarcinoma, malignancy level, alkalosis, Fenton reaction, hypoxia, tumor immune microenvironment

## Abstract

CCA is a highly desmoplastic malignant cancer and is the second most common primary liver malignancy after hepatocellular carcinoma (HCC), accounting for approximately 15% of all primary liver tumors. CCA has a poor prognosis, with an average five-year survival rate of 9%, which is lower than that of pancreatic cancer. Although considerable efforts have been invested into the genomics, epigenetics, and risk factors, very little is known about what might have been the key causes for the high malignancy level of CCA. In this review, we analyze the incidence and mortality of CCA in different regions based on data from 1994 to 2022 obtained from the International Agency for Research on Cancer (IARC), discuss the current status of treatment of the disease, and focus on what might be the main factors contributing to the high malignancy level of CCA: alkalosis caused by the Fenton reaction, hypoxia, and the TIME. The review includes studies published from 1979 to 2024, aiming to provide an updated synthesis of basic early classical theoretical knowledge and current knowledge about CCA. By revealing the epidemiological characteristics of CCA, the potential mechanisms of high malignancy, and the current challenges of treatment, this review aims to provide new directions for future cancer research, promote the development of personalized treatment strategies, and facilitate a deeper understanding and the more effective management of CCA worldwide.

## Simple Summary

This review analyzes the incidence and mortality of cholangiocarcinoma (CCA) in different regions around the world and focuses on the discussion of alkalosis caused by Fenton reactions, hypoxia, and tumor immune microenvironments (TIMEs), which may be the main factors contributing to the high malignancy level of CCA. This is of a certain significance for understanding the epidemiological characteristics of CCA and the potential mechanisms of high malignancy, thereby enhancing its global management and promoting its treatment strategies.

## 1. Etiology and Subtype of CCA

CCA originates from epithelial cells of bile ducts [1]. It is generally divided into three subtypes according to the anatomic site it is located in: intrahepatic CCA (iCCA), perihilar CCA (pCCA), and distal CCA (dCCA) [2,3]. In addition, a mixed HCC-CCA tumor is an independent subtype with the properties of iCCA and HCC [4,5]. CCA is highly malignant and is the second most common primary liver cancer after hepatocellular carcinoma, accounting for about 15% of all primary liver tumors [1,2,6,7].

This malignant tumor exhibits different epidemiological characteristics in different regions of the world, which is indicative of its unique risk factors and evolving etiological patterns in different populations. Studies have reported that liver fluke is the main risk factor for CCA in Southeast Asia [8]. Animal experiments have shown that C. sinensis and O. viverrini can cause CCA [9]. In the Korean Peninsula, the Russian Far East, northeastern and southern China, northern Vietnam, and Japan, C. sinensis is the main source of infection [10,11,12,13,14,15,16]. O. viverrini mainly infects the lower Mekong River region, where up to 100 million people are potentially at risk of CCA [17,18,19,20]. Studies in Asia have consistently shown that the hepatitis B virus (HBV) is also closely associated with the development of CCA, especially iCCA [21,22].

In western countries, primary sclerosing cholangitis is one of the main risk factors for CCA [23]. Primary sclerosing cholangitis is characterized by the bile duct structure and cholestasis due to chronic inflammation of the bile duct. Primary sclerosing cholangitis patients have a 400-fold higher risk of CCA than the general population, with an annual risk of 2% and a cumulative incidence of 20% over 30 years [24,25]. Choledochal cysts are cystic dilatations of the biliary system and are also established risk factors for CCA [26,27]. In a recent multicenter retrospective study involving 17 hepatobiliary centers in Germany, the incidence of CCA in patients with Caroli disease and Caroli syndrome was 7.1%. The overall risk of CCA appears to be higher in patients with Caroli disease and Caroli syndrome, both of which are genetic diseases characterized by cystic dilatation of the intrahepatic bile duct [28]. Liver cirrhosis, cholelithiasis, and choledocholithiasis have been identified as risk factors for CCA [29,30]. The direct carcinogenic effect of viruses on the liver cannot be underestimated. HBV and hepatitis C virus (HCV) depend on the presence of liver cirrhosis for carcinogenesis, and their characteristics and occurrence as a chronic viral infection may also be risk factors for the development of CCA; iCCA has a stronger tendency to develop [31,32]. Studies in western countries have shown that iCCA is more strongly associated with HCV than with HBV [33], a finding that was also confirmed by a Japanese study [22]. In addition, non-alcoholic fatty liver disease has a strong association with CCA and a positive correlation with iCCA [34]. Type 2 diabetes also increases the risk of CCA [35]. Other risk factors include inflammatory bowel disease, alcohol consumption, and smoking, and, of course, the controversial factors of obesity and hypertension [36], as seen in Table 1.

## 2. Geodemographic Distributions of Disease Incidence and Mortality

A large number of studies have reported that the incidence and mortality of CCA are still increasing year by year, and it has a high recurrence rate [2,37,38]. Based on the continuous updates and incomplete statistics of the IARC from 1994 to 2022, we summarized the incidence, mortality, and mortality-to-incidence ratio (MIR) of the globe, Europe, Asia, Northern America, Latin America and the Caribbean (LAC), Africa, and Oceania. We summarized the incidence, mortality, and MIR values of males and females and then processed the data and presented them (Supplementary Tables S1–S9).

**Figure 1:**
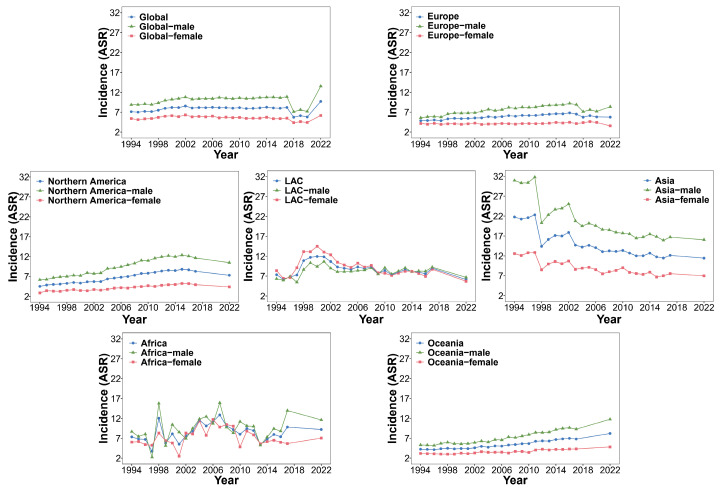
Trends in incidence for the glob and continents by cancer sites (liver, intrahepatic bile duct, gallbladder, etc.) from 1994 to 2022.

**Figure 2:**
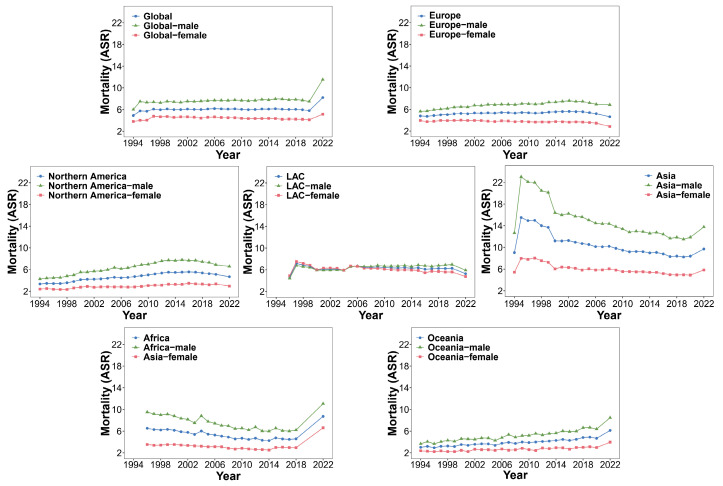
Trends in mortality in the globe and continents by cancer sites (liver, intrahepatic bile duct, gallbladder, etc.) from 1994 to 2022.

**Figure 3:**
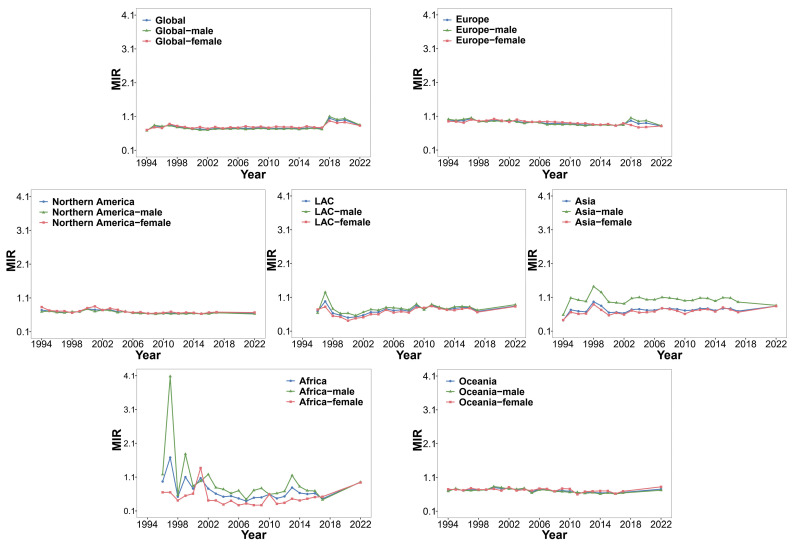
Trends in MIR in the globe and continents by cancer sites (liver, intrahepatic bile duct, gallbladder, etc.) from 1994 to 2022.

The trend charts of incidence, mortality, and MIR were constructed based on the data in Tables S1–S9 (Figure 1, Figure 2 and Figure 3). As can be seen from Figure 1, in the past 20 to 30 years, although the global incidence showed a downward trend from 2017 to 2018, it showed an overall upward trend before 2017 and after 2018. The incidence levels in Europe and Oceania were also on the rise. The incidence in Northern America showed a downward trend in 2015, but the downward trend was weak. The incidence in Asia showed a clear downward trend, but its Age-Standardized Rate per 100,000 (ASR) value of incidence was still higher than that in other continents. The incidence in LAC was basically stable except for a peak from 1998 to 2002. The incidence in Africa was very unstable, which also indirectly reflects that its basic medical level and public health status still face serious challenges [39]. Except for LAC, where the incidence in females was higher than that in males, the incidence in males in other continents was higher. Figure 2 shows that in the past 20 to 30 years, the mortality levels in the globe, Africa, and Oceania have been on an upward trend as a whole, reaching a new high in 2022. The mortality in Europe showed a downward trend in 2018. The overall mortality in Northern America showed a downward trend in 2017; the mortality of males also showed a downward trend in 2017, while the mortality of females remained stable. There was no obvious fluctuating trend in the mortality level in LAC. The mortality in Asia was declining overall, but the ASR value of the mortality was higher than that in other continents. According to Figure 3, it could be observed that the MIR indicators in the world and various continents were relatively stable, and the MIR values were generally less than 1, except in Asia and Africa. This partly reflects that CCA in Europe, Northern America, LAC, and Oceania may be more easily treated or controlled. In Africa and Asia, the MIR values showed greater fluctuations; specifically, the MIR of males was generally higher than that of females. These two regions may need to strengthen early screening and treatment strategies.

**Figure 4:**
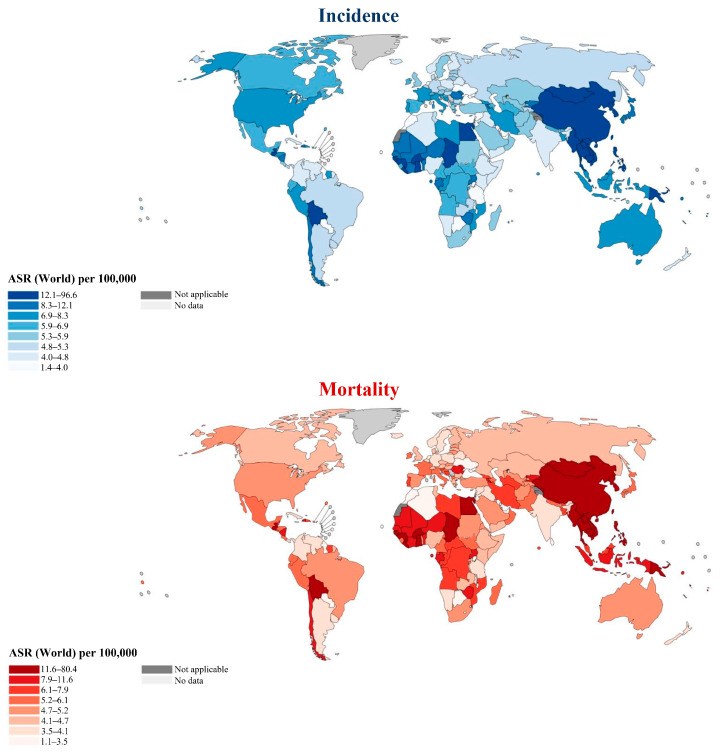
Incidence and mortality of cancer sites (liver, intrahepatic bile duct, gallbladder, etc.) by region of the world in 2022. Reprinted from IARC (https://gco.iarc.who.int/today (accessed on 24 December 2024)).

In 2022, the number of people who developed and died worldwide is alarming. As can be seen from Table 2 and Table 3, the incidence and mortality of CCA in Asia far exceed those in other continents, with Europe ranking second. This reflects that the epidemiology of CCA is diverse around the world [40]. Statistics from the IARC also show the status of CCA in each region or country in 2022 [41]. From the perspective of ASR, the global incidence of cancer sites from the liver, intrahepatic bile ducts, and gallbladder ranges from 1.4 to 96.6 per 100,000 people, while the mortality ranges from 1.1 to 80.4 per 100,000 people (Figure 4). Considering the incidence (mortality), the countries with higher ASR in Asia are Mongolia 96.6 (80.4), Lao People’s De. 26.3 (24.7), Cambodia 26.2 (24.5), Thailand 24.0 (23.0), Vietnam 20.4 (19.4), Korea, Republic. 16.3 (12.2), and China 16.2 (13.5). Moldova, which has the highest ASR in Europe, is 10.4 (9.3), and the United States of Americ., which has the highest ASR in Northern America, is 7.4 (4.7). In LAC, the country with the highest ASR is Guatemala at 15.9 (15.2). In Oceania, the countries with relatively high ASR are Guam at 15.1 (11.9) and Papua New Guinea at 13.3 (12.8). In Africa, the countries with relatively high ASR are Egypt at 32.9 (31.5), Guinea at 21.4 (20.9), Chad at 19.1 (18.6), The Republic of the Gam. at 17.9 (17.5), Burkina Faso at 17.5 (17.0), and Ghana at 16.2 (15.1).

The above data show that the epidemiological characteristics of CCA vary significantly worldwide, which may be related to local sanitary conditions, lifestyles, environmental factors, medical conditions, and the prevalence of screening. Therefore, strengthening early screening and treatment strategies, especially in areas with high incidence and mortality, is crucial for improving the prognosis of CCA patients.

## 3. State of Treatment of the Disease

Researchers have conducted in-depth studies on CCA from many perspectives, including risk of disease, pathogenic factors, classification, cell origin, genetic and epigenetic abnormalities, molecular changes, and biomarker discovery and treatment [1,23,40]. These studies have made people fully aware of it. However, the diagnosis, treatment, and prognosis of CCA still face huge challenges, which will directly affect the patient’s survival rate. The treatment of CCA usually depends on specific factors of the patient and the tumor, including age, physical condition, the patient’s underlying liver health, and the extent of the disease.

iCCA is a highly heterogeneous tumor [2], which makes it difficult to unify its treatment strategy, so patients often need targeted and personalized treatment strategies [42,43]. Surgery may be the only cure, but unfortunately, accurate diagnosis and staging of iCCA require a variety of imaging techniques and molecular tests, and the availability and accuracy of these technologies vary in different regions, resulting in many patients missing their best opportunity for surgery at the time of diagnosis. For early stage iCCA, liver transplantation may be a treatment option, but there is controversy about its applicability in advanced or vascular invasion tumors [42]. A combination chemotherapy of gemcitabine and cisplatin is the main treatment for advanced iCCA, but the treatment effect is limited [43,44].

PCCA is located in the central part of the liver, the tumor usually grows infiltratively, and it is closely related to the portal vessels, which makes surgical resection very difficult. In terms of radical surgery, deciding whether to perform extended radical surgery and/or vascular resection or reconstruction and determining how to balance radical resection with the quality of postoperative patient’s life and complication risk need to be resolved. The role of radiotherapy and chemotherapy in the treatment of pCCA is not yet fully understood, and how to select appropriate drugs and treatment regimens to improve treatment outcomes is a difficult problem [45].

The number of involved lymph nodes is very valuable for prognosis in dCCA [46]. Studies have reported that an increased number of involved lymph nodes is associated with a lower survival rate [47,48,49,50]. Approximately 370 patients were enrolled in the study at 24 hospitals in Japan between 2001 and 2010, and the number of involved lymph nodes was a strong predictor of survival in dCCA patients [51]. However, there are currently challenges in evaluating the status of lymph nodes. The detection of lymph node metastasis usually relies on pathological evaluation, and preoperative imaging studies (such as CT and PET) are not always accurate in evaluating lymph node metastasis [51,52,53].

Some researchers reviewed all 394 patients with histologically confirmed CCA who underwent surgical exploration at Johns Hopkins Hospital over a 23-year period and found that resection surgery improved survival. The 5-year survival rates for resected intrahepatic, perihilar, and distal tumors were 44%, 11%, and 28%, respectively, and the median survival rates were 26, 19, and 22 months, respectively. Postoperative radiation therapy did not improve survival [54]. Another study reported 564 CCA patients who underwent surgery between 1973 and 2004. The 5-year survival rates of resected intrahepatic, perihilar, and distal tumors were 63%, 30%, and 27%, respectively, and the median survival rates were 80, 30, and 25 months, respectively [55]. These clinical studies have suggested that resection should still be the main treatment method and that postoperative adjuvant radiotherapy has no effect on survival. Therefore, new drugs or strategies are needed to provide adjuvant therapy to improve survival [54]. Immunotherapy can be used as an adjuvant therapy after surgery to reduce the chance of cancer recurrence. Immunotherapy has dramatically revolutionized cancer treatment strategies over the past decade, but it is not the ultimate solution for cancer treatment. A major challenge to immunotherapy is the increased stiffness of the profibrotic extracellular matrix of tumors, which limits the penetration of immunomodulatory drugs and T cells, thereby affecting their engagement with tumor cells [56]. In contrast, tumor microenvironment (TME)-based therapeutic strategies mark the beginning of a new chapter in cancer treatment [57,58]. Many TME-based clinical trials are ongoing with some results, as shown in Table S10.

TME-based therapies use TME components, related pathways, and active molecules as therapeutic targets [59]. It has been suggested that TME component-based therapies combined with cytotoxic chemotherapy will produce optimal results [60]. In addition, blocking the communication between tumor cells and TME, such as inhibiting the release of specific cytokines, is considered a promising research approach. It has also been suggested by researchers that studies of TME-based reprogramming can help to gain insight into the process of cancer development and provide important information for the development of new therapeutic strategies [61]. Biomimetic nanocarriers exhibit lower clearance rates, lower immunogenicity, and higher targeting, showing great potential for application in cancer therapy [59,62,63]. Recent studies on TME-based biomimetic NPs are underway, including Erythrocyte Membrane-Coated NPs, Tumor Cell Membrane-Coated NPs, Immune Cell Membrane-Coated NPs, Proteins or Peptides-Based Biomimetic NPs, and Other Biomimetic NPs (Folic Acid, Monoclonal Antibodies, Tumor-Penetrating Peptides, Aptamers, etc.) [59].

Researchers are actively exploring the deep integration of computer science and technology methods such as machine learning and deep learning with multi-omics data in order to provide strong support for the adjuvant treatment of CCA. In the process, they successfully constructed risk prediction models, early diagnosis models, prognosis models, and survival rate prediction models that are closely related to CCA [64,65,66,67]. These models have begun to show potential in some specific cases; however, their universality in a wider range is still insufficient. The key direction of future research should focus on improving the accuracy and applicability of these models. This requires starting from the root cause, deeply exploring the key factors that contribute to the high malignancy level of CCA, and conducting systematic research on this basis in order to develop more targeted and effective adjuvant treatment plans for CCA patients.

In general, although surgical resection is the main treatment for CCA, surgical opportunities are often limited because most patients are already in the advanced stage of the disease when diagnosed. The effect of postoperative adjuvant therapy such as radiotherapy and chemotherapy is not ideal, and the application of immunotherapy in CCA is also limited by the TME. In recent years, TME-based therapy strategies and the combination of multi-omics data and machine learning have provided new ideas for the treatment of CCA, but the universality and effectiveness of these emerging methods still need to be further verified, as shown in Table 4. Therefore, future studies need to more deeply explore the biological characteristics of CCA and develop more targeted treatment options to improve patients’ prognosis and quality of life.

## 4. Factors Contributing to the High Malignancy Level of the Disease

### 4.1. Framework for Cancer Evolution Based on Fenton Reactions and Associated Metabolic Reprogramming

From the very beginning of cancer, all tumor cells will have alkaline intracellular and acidic extracellular components [68]. Studies have shown that solid tumor tissues tend to have (local) iron accumulation [69], and innate immune cells will appear at the site of inflammation and release a large amount of $H_{2}O_{2}$ at the same time. This phenomenon will lead to the Fenton reaction [70,71]:

(1) $${Fe}^{2+}+H_{2}O_{2}\to {Fe}^{3+}+OH^{-}+\cdot OH$$

The ·OH produced by this reaction will attract more immune cells again and then produce more $H_{2}O_{2}$, realizing a vicious cycle. During this cycle, $OH^{-}$ will be continuously produced; $OH^{-}$ cannot be directly pumped out of the cell because the cell does not allow charged molecules to continue moving in and out of the cell, which will destroy the electrical neutrality of the cell [72]. The continuous production of $OH^{-}$ causes the pH level in the tumor cell to rise, leading to alkalosis. It should be noted that in the Fenton reaction, ${Fe}^{2+}$ comes from iron–sulfur clusters [73], heme [74], and the labile iron pool [75]. There are various molecules such as NAD(P)H, superoxide (${\cdot O}_{2}^{-}$), $S^{2-}$, and ascorbic acid, which can reduce ${Fe}^{3+}$ to ${Fe}^{2+}$:

(2) $$H_{2}O_{2}+{\cdot O}_{2}^{-}\overset{{Fe}^{2+}}{\to }{OH}^{-}+\cdot OH+O_{2}$$

The Fenton reaction can occur repeatedly.

The proliferation of tumor cells may be driven by the Fenton reaction, which may also be a way for tumor cells to survive. Because the continuous production of $OH^{-}$ will change the pH of the cytoplasm and cause tumor cells to die, $OH^{-}$ needs to be neutralized [70,76]. The affected tumor cells synthesize nucleotides and consume ATP through glycolysis, which is accompanied by the production of $H^{+}$ [70]. The migration of tumor cells is related to the synthesis and accumulation of sialic acid, which may be caused by the Fenton reaction [77,78]. Sialic acid is a negatively charged nine-carbon sugar that accumulates at a high density on the surfaces of cancer cells during tumor development. As the charge density of sialic acid increases, it will produce stronger and stronger electrostatic repulsion, resulting in enhanced cell-to-cell adhesion, actomyosin contraction, protrusion, and migration [78,79]. Studies have shown that the continuous synthesis of sialic acid will produce more $H^{+}$, which also neutralizes the $OH^{-}$ produced by the Fenton reaction and relieves alkalosis [70,76,78].

Many metabolic changes have been observed in cancer compared to normal tissues. Some of these changes involve partial or substantial reorganization of normal metabolic processes, known as metabolic reprogramming [80,81]. The high degree of similarity in the behaviors of different tumors may be due to their use of several common metabolic reprogramming measures, including nucleotide and sialic acid synthesis, while the specificity of each tumor is due to their use of different combinations of metabolic reprogramming measures. It has been reported in the literature that the reprogrammed metabolism in cancer produces more $H^{+}$ than its original metabolism [72,77]. Cancers tend to upregulate protein genes that produce $H^{+}$ and downregulate protein genes that consume $H^{+}$. Cancers may overcome the constant alkalinization stress by reprogramming their metabolism at the whole cell level and find sustained ways to address the overproduction of other end products resulting from metabolic reprogramming.

Computational chemistry research has shown that the total rate of $H^{+}$ produced by all metabolic reprogramming measures is strongly positively correlated with the rate V(FR,$OH^{-}$) of $OH^{-}$ produced by the continuous Fenton reaction, and different tumors use different metabolic reprogramming combinations [72,77], which strongly indicates that the metabolic reprogramming of tumors is initiated under alkalization stress to maintain pH stability. Then, the sum of the rate of $OH^{-}$ produced by the Fenton reaction and the rate of $H^{+}$ produced by de novo nucleotide synthesis and sialic acid synthesis will almost reach a balance, resulting in the following formula:

(3) $$V(\mathrm{FR},OH^{-})=V(\mathrm{NS},H^{+})+V(\mathrm{SS},H^{+})+\varepsilon$$

**Figure 5:**
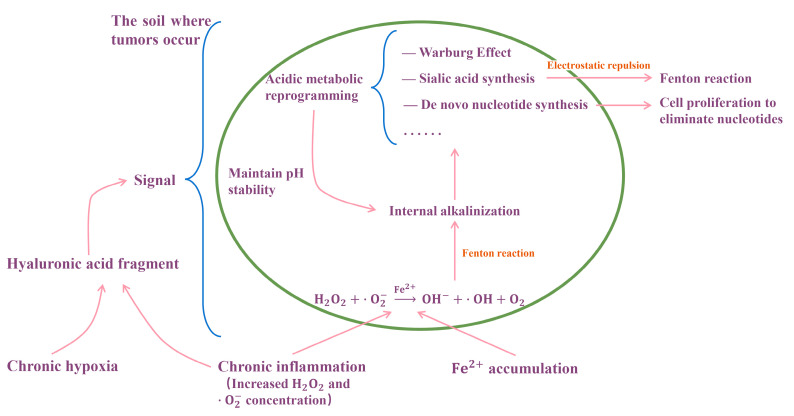
Model of the Fenton reaction driving tumor cell proliferation and cell migration. Chronic inflammation leads to local iron accumulation in the cytoplasm; then, the Fenton reaction occurs [70]. In order to maintain pH stability, cells initiate a series of acidification metabolic reprogramming [76]. In the precancerous stage, cells mainly maintain pH balance by upregulating acidic proteins, downregulating basic proteins, undergoing a large number of post-translational modifications, and synthesizing fatty acids. With the intensification of chronic inflammation and the strengthening of iron accumulation (the two form a vicious circle), the Fenton reaction continues to intensify, and cells begin to use stronger but more difficult to initiate acidification processes, including de novo nucleotide synthesis and sialic acid synthesis [77]. Cells produce some behaviors to provide an outlet for these reprogrammed metabolisms: cell proliferation, cell migration, or other [72].

Among them, V(NS,$H^{+}$) and V(SS,$H^{+}$) are the rates of $H^{+}$ produced by de novo nucleotide synthesis and sialic acid synthesis, respectively. ε is a relatively small number, representing the rate of $H^{+}$ produced by other reprogramming measures as a whole. As can be seen from Formula (3), V(NS,$H^{+}$) and V(SS,$H^{+}$) are complementary and negatively correlated. In addition, V(NS,$H^{+}$) is statistically strongly correlated with the rate of tumor cell proliferation, while V(SS,$H^{+}$) is statistically strongly correlated with the rate of tumor metastasis; these two statistical correlations give a strong indication that de novo nucleotide synthesis may drive cell proliferation [70], and sialic acid synthesis may drive cell metastasis [78]. The occurrence of cell proliferation, migration, and other phenomena may be due to the way cells overcome alkalosis and survive. Alkalosis generated by Fenton reaction is also universal in CCA. The model of tumor evolution to malignancy is shown in Figure 5.

### 4.2. Hypoxic Stress

Most solid tumors have a microenvironment characterized by hypoxia. The limited blood oxygen supply causes the developing tumors to usually live in a hypoxic environment [82]. The hypoxic environment can enhance the angiogenesis, proliferation, and invasion of tumor cells. Similarly, in CCA studies, hypoxia may directly induce the transcription of HIF1A and its protein level, thereby activating its downstream hypoxia signaling. Mechanistically, phosphatidylinositol 3-kinase/AKT and nuclear factor κB pathways have been shown to induce the expression of HIF1A mRNA, which is responsible for inducing hypoxia [83,84]. Hypoxia-induced vascular endothelial growth factor in tumors may trigger the polarization of cancer-associated fibroblasts and tumor-associated endothelial cells and promote tumor progression [85,86].

Hypoxic stress causes some metabolic reprogramming in CCA tumor cells, thereby inducing the polarization of tumor-associated macrophages toward the M2 phenotype and TIME remodeling in CCA [87,88,89]. Studies have found that hypoxia-dependent PPARγ-mediated oxidation of fatty acids in ${APOE}^{+}$ TAMs promote the polarization of macrophages toward the M2 phenotype by activating the HIF1A-PPARG-CD36 axis. These polarized macrophages recruit Treg cells through the CCL3-CCR5 pair, forming an immunosuppressive microenvironment and effectively inhibiting T cell activity [90].

**Figure 6:**
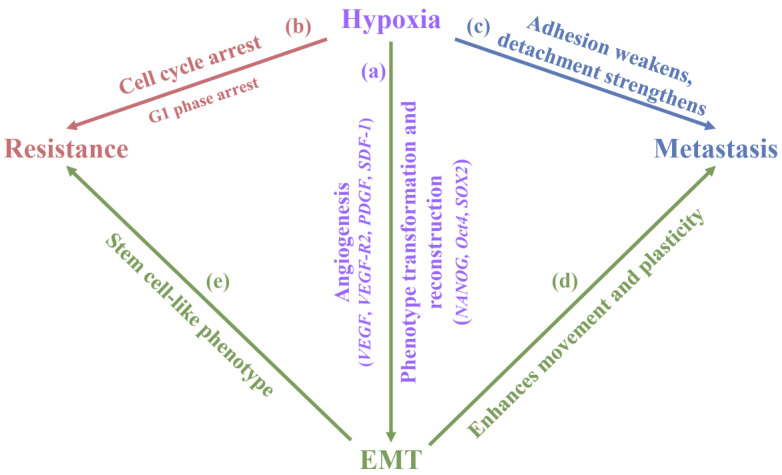
(a) Hypoxia promotes EMT by activating hypoxia-inducible factors. Hypoxia-inducible factors can upregulate EMT-related transcription factors (Snail, Slug, and Twist), which reduce the expression of epithelial cell markers and increase the expression of mesenchymal cell markers [101,102]. (b) The oxygen microenvironment can increase the resistance of CCA to drugs, chemotherapy, and radiotherapy. For example, arresting the cell cycle makes CCA cells insensitive to certain cycle-specific chemotherapeutic drugs [103]; promoting autophagy of CCA cells increases their tolerance to nutrient deficiency and oxidative stress, thereby enhancing the survival ability of CCA [104,105,106]. (c) Hypoxia promotes the degradation of the extracellular matrix by activating genes such as metalloproteinases, providing a physical channel for the invasion and metastasis of CCA. (d) EMT is a key step for CCA to acquire invasive and metastatic abilities. During the EMT process, epithelial cells lose their polarity characteristics and tight junctions between cells and acquire the characteristics of mesenchymal cells, including enhanced migration and invasion abilities. This process can also allow tumor cells to cross the basement membrane, enter blood vessels or lymphatic vessels, form circulating tumor cells, and then form metastases in distant organs [107]. (e) EMT-induced cell phenotypic changes may make it easier for tumor cells to evade the surveillance of the immune system, thereby increasing their ability to escape the immune system.

HIF1A induced by hypoxia is also involved in the transcription and translation of the Sonic Hedgehog pathway, leading to therapeutic resistance in CCA. Triggering of the Sonic Hedgehog pathway stimulates cancer stem cell transcription factors (NANOG, Oct4, and SOX2) and enhances their expression. This affects CD133 expression and initiates the epithelial–mesenchymal transition (EMT) process in CCA. Hypoxia leads to the downregulation of certain cell adhesion molecules and promotes tumor cell detachment and the expression of EMT markers (N-cadherin and Vimentin), which are acute events in cancer metastasis [91]. Hypoxia induces SKA3 expression through PARP1/HIF1A axis in CCA to enhance fatty acid synthesis [92] and induces the high expression of Rab1a by inhibiting miR-212-3p [93], leading to poor prognosis in CCA patients. Figure 6 shows that hypoxia plays an important role in the biological processes of EMT, metastasis, and resistance of CCA.

It is possible to solve the hypoxic stress issue by improving CCA angiogenesis, regulating hypoxia factors, and implementing nanotechnology and aerobic therapy. Inhibiting VEGF in CCA and then promoting healthy angiogenesis may improve the oxygen supply of CCA [94,95,96]. Increasing oxygen supply can also be achieved by using nanoparticles as drug carriers [97,98] to inhibit the activity of HIF1A and reduce the malignant phenotypes of tumors caused by hypoxia. Hyperbaric oxygen therapy can help improve the hypoxic state of the microenvironment of CCA and improve the effectiveness of radiotherapy and chemotherapy [99,100].

### 4.3. Macrophages and Neutrophils in TIMEs

The TIME has a significant impact on the occurrence, development, and response to treatment of tumors [108,109]. The high malignancy of CCA is related to the role of the TIME [110]. Job et al. found that the TIME in iCCA contains a variety of immune cells, which affect the progression of tumors through different mechanisms. They identified four immune subtypes through fine classification, each of which is associated with a specific immune escape mechanism and patient prognosis [111]. This shows that the mechanism of TIME is complex, some of which (along with their related cytokines) play an anti-tumor role, but others are pro-tumorigenic [112]. Bao et al. discovered the presence of a specific macrophage subtype, ${APOE}^{+}{C1QB}^{+}TAM$, in iCCA through proteomics, whole exome sequencing, and single-cell RNA sequencing. This macrophage subtype promotes the inflammatory response by affecting the activity state of ${CD4}^{+}$ T cells to secrete TNF-α, and ${APOE}^{+}{C1QB}^{+}TAM$ is associated with the poor prognosis of iCCA [113].

**Figure 7:**
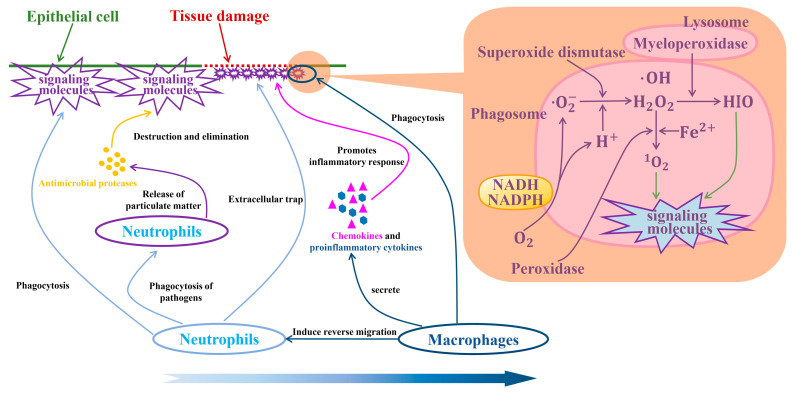
Neutrophils and macrophages repair the damaged sites of bile duct epithelial cells [124,125]. The mechanism on the right side of the figure is one of the sterilization methods of macrophages: the oxygen-dependent system [126,127]. During phagocytosis, the reductase I (NADH), reductase II (NADPH), and molecular oxygen ($O_{2}$) on the cell membrane are activated to generate superoxide anions ($O_{2}^{-}$), free hydroxyl radicals (OH), hydrogen peroxide (${H_{2}O}_{2}$), and singlet oxygen (^1^$O_{2}$), thereby producing a sterilizing effect.

Chen et al. used multi-omics analysis, including whole exome sequencing, bulk and single-cell RNA sequencing, methylation microarrays, and multiplex immunostaining, and found that although iCCA showed high heterogeneity at the genomic level, the TIME had relatively low heterogeneity among different iCCA tumors. This suggests that immune cells in TIMEs, including macrophages and neutrophils, may play a consistent role in the progression of iCCA [114]. Figure 7 shows that when the epithelial cells of the bile duct are damaged, neutrophils will first appear and engulf signaling molecules, such as chemical factors, biological factors, and metabolites. Under the influence of departiculation, neutrophils will release antimicrobial proteases to destroy and eliminate signaling molecules. Subsequently, macrophages can induce neutrophils to leave the damaged site of the tissue in a reverse migration manner, thereby promoting the recovery of inflammation.

When macrophages and neutrophils undergo polarization and tissue repair occurs, high metabolic oxygen consumption leads to local hypoxia, which ultimately enhances the high malignancy of CCA. Perhaps we can focus on drugs or immunomodulators to change the polarization states of macrophages and neutrophils. For example, we can start with immune checkpoint inhibitors (PD-1/PD-L1) [115] or cytokines (such as IFN-γ or LPS) to promote the polarization of macrophages toward the M1 phenotype and neutrophils toward the N1 phenotype [116,117]. This can change the pro-tumor type to the anti-tumor type. The key signaling pathways of pro-tumor polarization may also be a breakthrough. Regarding the intervention of signaling pathways, Cho et al. proposed through proteomic integrated analysis that the TIME-based stem-like subtype [118] may be involved in tumor stemness-related signaling pathways or in inhibiting the PI3K-Akt pathway and the JAK-STAT pathway, among other pathways, which may help reduce the pro-tumoral activity of M2-TAM and N2 [119]. Of course, we can also try to induce apoptosis of macrophages and neutrophils to reduce their number and influence in the TIME [120,121]. Intervention from the perspective of metabolic pathways, inhibiting the glycolytic pathway, may reduce the metabolic activity of these cells [122,123], thereby alleviating local hypoxia. In the future, more innovative treatments may be developed to address the challenges posed by the polarization of macrophages and neutrophils and by local hypoxia.

The high malignancy of CCA is the result of the combined action of multiple factors. The Fenton reaction and its induced metabolic reprogramming promote cell proliferation and migration by maintaining intracellular acid–base balance. The hypoxic environment promotes tumor cell invasion and immune escape by activating HIF1A and its downstream signaling pathways. Macrophages and neutrophils in the TIME further aggravate the malignant behavior of the tumor through their polarization state and metabolic activity. These mechanisms interact with each other and jointly promote the high malignant progression of CCA. Subsequent studies can explore the interactions between these mechanisms, reveal their specific roles in the progression of CCA, and provide a theoretical basis for the development of new therapeutic targets and intervention strategies.

## 5. Conclusions

This review focuses on the highly malignant characteristics of CCA and its epidemiological characteristics. It attempts to provide a reference for understanding the epidemiological characteristics, potential pathogenic mechanisms, and treatment challenges of CCA by analyzing the incidence and mortality in different regions around the world, combined with the etiology, subtypes, treatment status, and possible factors that contribute to its high malignancy level (such as alkalosis produced by the Fenton reaction, hypoxic stress, and the roles of macrophages and neutrophils in the TIME).

The results suggest that the epidemiological characteristics of CCA vary significantly worldwide, which may be closely related to regional sanitary conditions, lifestyle, environmental factors, and the prevalence of screening. In addition, the Fenton reaction and its induced metabolic reprogramming, hypoxic environment, and the polarization state of macrophages and neutrophils in the TIME may be important factors that promote the high malignancy of CCA.

Although existing studies have extensively explored the pathogenesis, treatment strategies, and prognostic factors of CCA, there are still limitations. For example, the specific mechanism of the Fenton reaction in tumor cells has not been fully clarified, and its interaction with metabolic reprogramming still needs further study. In addition, the specific contributions of the adaptive changes of tumor cells under hypoxia conditions and the polarization states of macrophages and neutrophils in the TIME to the progression of CCA are still unclear. These limitations suggest that future studies need to explore the biological characteristics of CCA in more depth, reveal the molecular mechanisms of its highly malignant characteristics, and develop more targeted treatment strategies.

Future research directions should focus on the following aspects: (1) in-depth study of the interaction between the Fenton reaction and metabolic reprogramming and exploration of its potential mechanism in the occurrence and development of CCA; (2) analysis of the adaptive changes of tumor cells under hypoxia and their potential impact on treatment; and (3) development of new treatment strategies for macrophages and neutrophils in the TIME, such as changing their polarization state through immunomodulators or targeted intervention of related signaling pathways to reduce their pro-tumor activity. In addition, the adjuvant therapy model combining multi-omics data and machine learning technology also provides new ideas for the precise treatment of CCA, but its universality and accuracy still need to be further verified.

In summary, this review attempts to reveal the potential mechanisms of CCA’s high malignancy through analysis of its epidemiology, etiology, and treatment status, and it points out the limitations of existing research. Future research should be devoted to further exploring the biological characteristics of CCA and developing more targeted treatment strategies to improve patient prognosis and quality of life.

## Figures and Tables

**Table 1:** Summary of CCA risk factors from East Asian and Western countries [8,9,21,22,23,26,27,28,29,30,33,34,35,36].

East Asian Countries	Western Countries
*C. sinensis*	Primary sclerosing cholangitis
*O. viverrini*	Choledochal cysts
Hepatitis B virus	Caroli disease
	Caroli syndrome
	Liver cirrhosis
	Cholelithiasis
	Choledocholithiasis
	Hepatitis C virus
	Non-alcoholic fatty liver disease
	Type 2 diabetes mellitus
	Inflammatory bowel disease
	Alcohol consumption
	Smoking
	Obesity
	Hypertension

**Table 2:** Absolute numbers of cancer sites (liver, intrahepatic bile duct, gallbladder, etc.) by continents in 2022 with respect to incidence [41].

Continent	Both Sexes	Males	Females
Globe	988,627	644,214	344,413
Europe	101,541|10.27%	62,160|9.65%	39,381|11.43%
Asia	695,473|70.35%	468,793|72.77%	226,680|65.82%
Northern America	53,714|5.43%	36,007|5.59%	17,707|5.14%
LAC	53,203|5.38%	25,936|4.02%	27,267|7.92%
Africa	79,356|8.03%	47,715|7.41%	31,641|9.19%
Oceania	5340|0.54%	3603|0.56%	1737|0.50%

Reprinted from IARC (https://gco.iarc.who.int/today (accessed on 24 December 2024)) and Ref. [41].

**Table 3:** Absolute numbers of cancer sites (liver, intrahepatic bile duct, gallbladder, etc.) by continents in 2022 with respect to mortality [41].

Continents	Both Sexes	Males	Females
Globe	847,780	553,232	294,548
Europe	87,294|10.30%	54,060|9.77%	33,234|11.28%
Asia	597,749|70.51%	403,784|72.98%	193,965|65.85%
Northern America	37,528|4.42%	24,346|4.40%	13,182|4.48%
LAC	46,398|5.47%	23,215|4.20%	23,183|7.87%
Africa	74,513|8.79%	45,022|8.14%	29,491|10.01%
Oceania	4298|0.51%	2805|0.51%	1493|0.51%

Reprinted from IARC (https://gco.iarc.who.int/today (accessed on 24 December 2024)) and Ref. [41].

**Table 4:** Summary of the current status of CCA therapy [42,43,44,45,54,55,56,57,58,59,64,65,66,67].

Treatment	Applicable Situation	Effect	Challenge
Surgery	Early-stage CCA; No distant metastasis	5-year survival rate: intrahepatic 44–63%, perihilar 11–30%, distal 27–28%	Most patients are already in the advanced stage when diagnosed, and the chances of surgery are limited.
Liver transplantation	Early-stage iCCA	Possible treatment options	Applicability in advanced or vascular invasion tumors is controversial
Chemotherapy	Advanced CCA	Gemcitabine combined with cisplatin is the main treatment option, but the effect is limited	Limited efficacy in advanced iCCA; The role of pCCA in treatment is still unclear
Radiotherapy	Postoperative adjuvant therapy	No significant improvement in survival rate	The role of pCCA in treatment is still unclear
Immunotherapy	Postoperative adjuvant therapy	Reduce the risk of recurrence	Limited penetration of drugs and T cells due to TME restrictions
TME-based therapy	Targeting TME	Some clinical trials have achieved results, but their universality and effectiveness need further verification	Development and validation of therapy strategies is ongoing
Machine (deep) learning	Adjunctive therapy	Constructed risk prediction, early diagnosis, prognosis and survival prediction models	The universality and accuracy of the model needs to be improved

## Data Availability

Not applicable.

## Supplementary Materials

The following supporting information can be downloaded at https://www.mdpi.com/article/10.3390/biology14040351/s1.

## Abbreviations

CCA, cholangiocarcinoma; TIME, tumor immune microenvironment; HCC, hepatocellular carcinoma; IARC, International Agency for Research on Cancer; iCCA, intrahepatic CCA; pCCA, perihilar CCA; dCCA, distal CCA; HBV, hepatitis B virus; HCV, hepatitis C virus; MIR, mortality-to-incidence ratio; LAC, Latin America and the Caribbean; ASR, Age-Standardized Rate per 100,000; TME, tumor microenvironment; and EMT, epithelial-mesenchymal transition.
