# Supplementary material for: Factors Contributing to the High Malignancy Level of Cholangiocarcinoma and Its Epidemiology: Literature Review and Data
Source: Biology (Basel). 2025 Mar 28;14(4):351. doi: 10.3390/biology14040351 (PMC12025278; doi:10.3390/biology14040351)
Supplement: Supplementary file 1 [file biology-14-00351-s001.zip › biology-3459880-supplementary.pdf]

Supplementary

Combined with the International Agency for Research on Cancer's (IARC) consideration of cancer sites (liver, intrahepatic bile duct, and gallbladder), and with continuous updates and incomplete statistics from 1994 to 2022, we will take the average of the incidence (or mortality) of the countries that are included in the statistics and belong to the same region, which roughly represents the incidence (or mortality) of the region. We summarized the incidence, mortality, and mortality-to-incidence ratio (MIR) of the global, Europe, Asia, Northern America, Latin America and the Caribbean (LAC), Africa, and Oceania, and summarized the incidence, mortality, and MIR of male and female respectively (Table S1-S9).

TME-based studies have the potential to provide new strategies and methods for the treatment of CCA, which has certain potential research value. Therefore, we also focused on some TME-based clinical trials, as shown in Table S10.

Table S1. IARC statistics on the ASR of the incidence of cancer sites (liver, intrahepatic bile duct, and gallbladder, etc.) in both sexes from 1994 to 2022, Ref [41].

Time	Global	Europe	Asia	Northern America	LAC	Africa	Oceania
1994	7.13	4.86	21.77	4.52	7.35	7.36	4.24
1995	7.01	4.91	21.26	4.85	6.20	6.82	4.20
1996	7.20	5.04	21.59	5.01	6.79	6.73	4.13
1997	7.16	4.86	22.32	5.09	7.28	3.75	4.36
1998	7.50	5.31	14.40	5.27	10.89	12.04	4.48
1999	7.98	5.45	16.13	5.49	11.72	5.77	4.32
2000	8.18	5.36	17.12	5.34	11.94	8.12	4.41
2001	8.16	5.43	16.99	5.66	11.85	5.53	4.39
2002	8.56	5.55	17.88	5.71	10.66	7.63	4.60
2003	8.05	5.57	14.70	5.72	9.29	8.79	4.96
2004	8.16	5.87	14.21	6.38	8.96	11.60	4.75
2005	8.13	5.71	14.65	6.60	8.67	10.10	5.07
2006	8.22	5.89	14.05	6.81	9.31	11.22	5.03
2007	8.13	6.13	13.05	7.00	8.89	12.87	5.30
2008	8.14	5.98	13.26	7.32	9.37	10.12	5.42
2009	8.01	6.19	13.12	7.75	7.67	9.21	5.62
2010	8.14	6.17	13.36	7.82	8.42	8.02	5.66
2011	7.93	6.20	12.70	8.05	7.34	9.47	6.24
2012	7.98	6.37	12.01	8.37	8.00	8.94	6.34
2013	8.08	6.49	12.03	8.56	8.63	5.45	6.30
2014	8.25	6.60	12.69	8.48	8.14	6.74	6.69
2015	8.07	6.58	11.75	8.81	7.98	7.97	6.84
2016	8.00	6.83	11.46	8.67	7.59	7.40	6.97
2017	8.19	6.51	12.13	8.30	9.00	9.83	6.80
2018	5.74	5.74	-	-	-	-	-
2019	6.13	6.13	-	-	-	-	-
2020	5.81	5.81	-	-	-	-	-
2022	9.72	5.75	11.43	7.31	6.14	9.22	8.20

The raw data were extracted from IARC (https://gco.iarc.who.int/overtime accessed on 24 December 2024) and data processed to generate Table S1. In the Table S1, "-" means no statistics are displayed.

Table S2. IARC statistics on the ASR of the incidence of cancer sites (liver, intrahepatic bile duct, and gallbladder, etc.) in males from 1994 to 2022, Ref [41].

Time	Global	Europe	Asia	Northern America	LAC	Africa	Oceania
1994	8.87	5.57	21.77	6.17	6.33	8.67	5.31
1995	8.89	5.84	21.26	6.26	5.98	7.47	5.28
1996	9.08	5.89	21.59	6.70	6.93	8.04	5.17

1997	8.92	5.77	22.32	6.90	5.45	2.25	5.70
1998	9.34	6.55	14.40	7.02	8.64	15.77	5.97
1999	9.96	6.80	16.13	7.28	10.32	5.14	5.63
2000	10.22	6.76	17.12	7.21	9.39	10.43	5.57
2001	10.43	6.79	16.99	7.92	10.65	8.55	5.66
2002	10.80	6.84	17.88	7.71	8.97	6.93	5.91
2003	10.26	7.21	14.70	7.88	8.09	9.48	6.29
2004	10.37	7.71	14.21	8.99	8.12	11.85	6.03
2005	10.40	7.39	14.65	9.13	8.17	12.45	6.65
2006	10.41	7.63	14.05	9.45	8.39	10.70	6.56
2007	10.68	8.18	13.05	9.92	8.53	15.91	7.32
2008	10.52	7.96	13.26	10.3	9.06	9.77	7.15
2009	10.39	8.25	13.12	11.04	7.52	8.37	7.55
2010	10.59	8.20	13.36	10.98	9.10	11.19	7.91
2011	10.43	8.29	12.70	11.59	7.50	10.12	8.44
2012	10.49	8.60	12.01	11.94	8.24	10.01	8.43
2013	10.67	8.74	12.03	12.21	9.06	5.25	8.54
2014	10.76	8.79	12.69	11.97	8.07	7.31	9.19
2015	10.78	8.87	11.75	12.37	8.29	9.42	9.49
2016	10.62	9.20	11.46	12.12	8.23	8.80	9.65
2017	10.88	8.89	12.13	11.66	9.25	13.97	9.29
2018	7.11	7.11	-	-	-	-	-
2019	7.62	7.62	-	-	-	-	-
2020	7.19	7.19	-	-	-	-	-
2022	13.54	8.36	16.05	10.48	6.69	11.63	11.76

The raw data were extracted from IARC (https://gco.iarc.who.int/overtime accessed on 24 December 2024) and data processed to generate Table S2. In the Table S2, "-" means no statistics are displayed.

Table S3. IARC statistics on the ASR of the incidence of cancer sites (liver, intrahepatic bile duct, and gallbladder, etc.) in females from 1994 to 2022, Ref [41].

Time	Global	Europe	Asia	Northern America	LAC	Africa	Oceania
1994	5.40	4.15	12.59	2.88	8.37	6.05	3.18
1995	5.13	3.98	12.09	3.43	6.42	6.17	3.12
1996	5.32	4.19	12.79	3.32	6.65	5.42	3.09
1997	5.40	3.95	12.85	3.28	9.11	5.25	3.03
1998	5.71	4.07	8.51	3.53	13.14	8.31	3.00
1999	5.99	4.09	9.95	3.70	13.12	6.40	3.01
2000	6.13	3.96	10.57	3.47	14.5	5.82	3.24
2001	5.90	4.08	10.03	3.41	13.04	2.52	3.13
2002	6.31	4.26	10.71	3.71	12.35	8.32	3.30
2003	5.84	3.93	8.62	3.56	10.49	8.09	3.63
2004	5.94	4.03	8.92	3.78	9.80	11.36	3.47
2005	5.85	4.03	9.12	4.07	9.17	7.74	3.49
2006	6.02	4.15	8.54	4.18	10.24	11.75	3.51
2007	5.58	4.09	7.49	4.08	9.25	9.83	3.27
2008	5.75	4.00	8.01	4.35	9.68	10.46	3.70
2009	5.63	4.13	8.33	4.46	7.81	10.04	3.68
2010	5.68	4.14	9.02	4.67	7.74	4.85	3.42
2011	5.44	4.11	7.87	4.51	7.17	8.81	4.05
2012	5.48	4.14	7.57	4.79	7.76	7.86	4.24
2013	5.50	4.24	7.34	4.92	8.20	5.64	4.05
2014	5.73	4.41	7.91	4.99	8.21	6.18	4.20

2015	5.36	4.28	6.69	5.24	7.68	6.51	4.19
2016	5.38	4.45	7.01	5.22	6.95	6.00	4.29
2017	5.50	4.14	7.54	4.94	8.74	5.69	4.31
2018	4.37	4.37	-	-	-	-	-
2019	4.64	4.64	-	-	-	-	-
2020	4.42	4.42	-	-	-	-	-
2022	6.18	3.55	6.99	4.39	5.70	7.05	4.82

The raw data were extracted from IARC (https://gco.iarc.who.int/overtime accessed on 24 December 2024) and data processed to generate Table S3. In the Table S3, "-" means no statistics are displayed.

Table S4. IARC statistics on the ASR of the mortality of cancer sites (liver, intrahepatic bile duct, and gallbladder, etc.) in both sexes from 1994 to 2022, Ref [41].

Time	Global	Europe	Asia	Northern America	LAC	Africa	Oceania
1994	4.90	4.80	9.04	3.34	-	-	3.02
1995	5.75	4.73	15.50	3.46	-	-	3.20
1996	5.69	4.87	14.94	3.42	4.66	6.53	2.94
1997	6.08	5.00	14.99	3.43	7.15	6.30	3.21
1998	5.96	5.04	14.00	3.57	6.87	6.22	3.26
1999	6.11	5.18	13.69	3.80	6.63	6.33	3.17
2000	5.99	5.25	11.20	4.14	5.96	6.17	3.53
2001	5.99	5.19	11.18	4.22	6.11	5.90	3.39
2002	6.09	5.34	11.26	4.23	6.12	5.78	3.58
2003	6.03	5.31	10.94	4.28	6.12	5.40	3.66
2004	6.00	5.36	10.71	4.40	5.90	6.03	3.65
2005	6.10	5.31	10.53	4.58	6.62	5.45	3.38
2006	6.18	5.42	10.14	4.48	6.64	5.29	3.76
2007	6.12	5.39	10.11	4.56	6.45	5.08	3.93
2008	6.08	5.31	10.2	4.71	6.43	4.91	3.73
2009	6.13	5.42	9.82	4.88	6.52	4.57	4.00
2010	6.04	5.37	9.47	5.02	6.40	4.70	3.90
2011	5.97	5.31	9.18	5.20	6.35	4.47	4.00
2012	6.01	5.36	9.24	5.36	6.32	4.71	4.10
2013	6.10	5.50	9.21	5.52	6.38	4.32	4.17
2014	6.08	5.55	8.99	5.47	6.28	4.26	4.29
2015	6.15	5.60	9.07	5.53	6.37	4.77	4.47
2016	6.06	5.63	8.79	5.58	6.10	4.57	4.30
2017	6.02	5.58	8.31	5.52	6.18	4.49	4.49
2018	6.04	5.56	8.39	5.38	6.24	4.59	4.83
2019	5.96	5.40	8.23	5.25	6.23	-	4.90
2020	5.80	5.21	8.38	5.12	6.28	-	4.68
2022	8.20	4.64	9.71	4.68	5.28	8.73	6.14

The raw data were extracted from IARC (https://gco.iarc.who.int/overtime accessed on 24 December 2024) and data processed to generate Table S4. In the Table S4, "-" means no statistics are displayed.

Table S5. IARC statistics on the ASR of the mortality of cancer sites (liver, intrahepatic bile duct, and gallbladder, etc.) in males from 1994 to 2022, Ref [41].

Time	Global	Europe	Asia	Northern America	LAC	Africa	Oceania
1994	6.00	5.64	12.66	4.27	-	-	3.67
1995	7.49	5.70	23.01	4.42	-	-	4.08
1996	7.33	5.94	22.08	4.47	4.42	9.52	3.66
1997	7.39	6.04	21.96	4.52	6.79	9.19	4.06
1998	7.26	6.17	20.46	4.81	6.57	9.00	4.30

1999	7.51	6.40	20.13	5.00	6.44	9.14	4.12
2000	7.41	6.47	16.38	5.53	5.92	8.80	4.60
2001	7.33	6.43	15.98	5.54	5.92	8.35	4.55
2002	7.53	6.74	16.23	5.71	5.93	8.18	4.49
2003	7.47	6.70	15.73	5.76	5.97	7.52	4.74
2004	7.57	6.90	15.61	5.98	5.88	8.82	4.73
2005	7.62	6.86	15.03	6.37	6.55	7.78	4.28
2006	7.71	6.94	14.46	6.14	6.65	7.44	4.80
2007	7.70	6.93	14.35	6.33	6.58	7.04	5.37
2008	7.68	6.88	14.36	6.62	6.57	6.97	4.89
2009	7.76	7.05	13.82	6.88	6.79	6.45	5.17
2010	7.68	7.03	13.40	6.98	6.67	6.55	5.20
2011	7.60	6.97	12.81	7.26	6.69	6.23	5.56
2012	7.68	7.04	12.98	7.58	6.70	6.77	5.29
2013	7.86	7.33	12.90	7.73	6.79	6.03	5.56
2014	7.78	7.35	12.59	7.66	6.61	6.00	5.64
2015	7.96	7.48	12.76	7.79	6.86	6.56	6.03
2016	7.93	7.60	12.41	7.68	6.74	6.09	5.91
2017	7.79	7.45	11.66	7.69	6.63	6.01	5.99
2018	7.85	7.46	11.86	7.44	6.78	6.22	6.63
2019	7.72	7.21	11.50	7.31	6.88	-	6.68
2020	7.48	6.95	11.87	6.89	6.96	-	6.39
2022	11.53	6.85	13.76	6.59	5.92	11.07	8.48

The raw data were extracted from IARC (https://gco.iarc.who.int/overtime accessed on 24 December 2024) and data processed to generate Table S5. In the Table S5, "-" means no statistics are displayed.

Table S6. IARC statistics on the ASR of the mortality of cancer sites (liver, intrahepatic bile duct, and gallbladder, etc.) in females from 1994 to 2022, Ref [41].

Time	Global	Europe	Asia	Northern America	LAC	Africa	Oceania
1994	3.80	3.95	5.41	2.40	-	-	2.37
1995	4.02	3.75	7.98	2.50	-	-	2.31
1996	4.04	3.81	7.80	2.37	4.90	3.54	2.22
1997	4.77	3.96	8.02	2.34	7.51	3.40	2.35
1998	4.66	3.92	7.54	2.34	7.17	3.43	2.23
1999	4.71	3.96	7.25	2.61	6.82	3.52	2.22
2000	4.57	4.02	6.03	2.76	6.00	3.54	2.47
2001	4.65	3.95	6.38	2.89	6.29	3.44	2.23
2002	4.65	3.95	6.29	2.74	6.31	3.38	2.68
2003	4.60	3.93	6.15	2.81	6.26	3.28	2.58
2004	4.43	3.83	5.82	2.82	5.92	3.24	2.57
2005	4.59	3.76	6.04	2.80	6.69	3.12	2.47
2006	4.64	3.90	5.83	2.81	6.64	3.14	2.72
2007	4.54	3.86	5.86	2.78	6.31	3.12	2.49
2008	4.49	3.73	6.04	2.80	6.28	2.85	2.56
2009	4.49	3.78	5.82	2.89	6.25	2.70	2.84
2010	4.39	3.71	5.54	3.06	6.13	2.85	2.59
2011	4.34	3.66	5.54	3.13	6.02	2.72	2.44
2012	4.35	3.69	5.49	3.13	5.94	2.65	2.90
2013	4.35	3.66	5.51	3.30	5.98	2.61	2.78
2014	4.38	3.75	5.39	3.28	5.95	2.52	2.94
2015	4.35	3.72	5.38	3.28	5.87	2.99	2.92
2016	4.20	3.65	5.18	3.48	5.45	3.05	2.68

2017	4.26	3.70	4.97	3.35	5.73	2.97	2.98
2018	4.23	3.66	4.92	3.33	5.69	2.96	3.02
2019	4.20	3.59	4.95	3.20	5.59	-	3.13
2020	4.12	3.46	4.89	3.35	5.60	-	2.98
2022	5.15	2.86	5.84	2.96	4.75	6.63	3.97

The raw data were extracted from IARC (https://gco.iarc.who.int/overtime accessed on 24 December 2024) and data processed to generate Table S6. In the Table S6, "-" means no statistics are displayed.

Table S7. MIR of cancer sites (liver, intrahepatic bile duct, and gallbladder, etc.) in both sexes from 1994 to 2022.

Time	Global	Europe	Asia	Northern America	LAC	Africa	Oceania
1994	0.69	0.99	0.42	0.74	-	-	0.71
1995	0.82	0.96	0.73	0.71	-	-	0.76
1996	0.79	0.97	0.69	0.68	0.69	0.97	0.71
1997	0.85	1.03	0.67	0.67	0.98	1.68	0.74
1998	0.79	0.95	0.97	0.68	0.63	0.52	0.73
1999	0.77	0.95	0.85	0.69	0.57	1.10	0.73
2000	0.73	0.98	0.65	0.78	0.50	0.76	0.80
2001	0.73	0.96	0.66	0.75	0.52	1.07	0.77
2002	0.71	0.96	0.63	0.74	0.57	0.76	0.78
2003	0.75	0.95	0.74	0.75	0.66	0.61	0.74
2004	0.74	0.91	0.75	0.69	0.66	0.52	0.77
2005	0.75	0.93	0.72	0.69	0.76	0.54	0.67
2006	0.75	0.92	0.72	0.66	0.71	0.47	0.75
2007	0.75	0.88	0.77	0.65	0.73	0.39	0.74
2008	0.75	0.89	0.77	0.64	0.69	0.49	0.69
2009	0.77	0.88	0.75	0.63	0.85	0.50	0.71
2010	0.74	0.87	0.71	0.64	0.76	0.59	0.69
2011	0.75	0.86	0.72	0.65	0.87	0.47	0.64
2012	0.75	0.84	0.77	0.64	0.79	0.53	0.65
2013	0.75	0.85	0.77	0.64	0.74	0.79	0.66
2014	0.74	0.84	0.71	0.65	0.77	0.63	0.64
2015	0.76	0.85	0.77	0.63	0.80	0.60	0.65
2016	0.76	0.82	0.77	0.64	0.80	0.62	0.62
2017	0.74	0.86	0.69	0.67	0.69	0.46	0.66
2018	1.05	0.97	-	-	-	-	-
2019	0.97	0.88	-	-	-	-	-
2020	1.00	0.90	-	-	-	-	-
2022	0.84	0.81	0.85	0.64	0.86	0.95	0.75

In the Table S7, "-" means no statistics are displayed.

Table S8. MIR of cancer sites (liver, intrahepatic bile duct, and gallbladder, etc.) in males from 1994 to 2022.

Time	Global	Europe	Asia	Northern America	LAC	Africa	Oceania
1994	0.68	1.01	0.58	0.69	-	-	0.69
1995	0.84	0.98	1.08	0.71	-	-	0.77
1996	0.81	1.01	1.02	0.67	0.64	1.18	0.71
1997	0.83	1.05	0.98	0.66	1.25	4.08	0.71
1998	0.78	0.94	1.42	0.69	0.76	0.57	0.72
1999	0.75	0.94	1.25	0.69	0.62	1.78	0.73
2000	0.73	0.96	0.96	0.77	0.63	0.84	0.83
2001	0.70	0.95	0.94	0.70	0.56	0.98	0.80
2002	0.70	0.99	0.91	0.74	0.66	1.18	0.76
2003	0.73	0.93	1.07	0.73	0.74	0.79	0.75

2004	0.73	0.89	1.10	0.67	0.72	0.74	0.78
2005	0.73	0.93	1.03	0.70	0.80	0.62	0.64
2006	0.74	0.91	1.03	0.65	0.79	0.70	0.73
2007	0.72	0.85	1.10	0.64	0.77	0.44	0.73
2008	0.73	0.86	1.08	0.64	0.73	0.71	0.68
2009	0.75	0.85	1.05	0.62	0.90	0.77	0.68
2010	0.73	0.86	1.00	0.64	0.73	0.59	0.66
2011	0.73	0.84	1.01	0.63	0.89	0.62	0.66
2012	0.73	0.82	1.08	0.63	0.81	0.68	0.63
2013	0.74	0.84	1.07	0.63	0.75	1.15	0.65
2014	0.72	0.84	0.99	0.64	0.82	0.82	0.61
2015	0.74	0.84	1.09	0.63	0.83	0.70	0.64
2016	0.75	0.83	1.08	0.63	0.82	0.69	0.61
2017	0.72	0.84	0.96	0.66	0.72	0.43	0.64
2018	1.10	1.05	-	-	-	-	-
2019	1.01	0.95	-	-	-	-	-
2020	1.04	0.97	-	-	-	-	-
2022	0.85	0.82	0.86	0.63	0.88	0.95	0.72

In the Table S8, "-" means no statistics are displayed.

Table S9. MIR of cancer sites (liver, intrahepatic bile duct, and gallbladder, etc.) in females from 1994 to 2022.

Time	Global	Europe	Asia	Northern America	LAC	Africa	Oceania
1994	0.70	0.95	0.43	0.83	-	-	0.75
1995	0.78	0.94	0.66	0.73	-	-	0.74
1996	0.76	0.91	0.61	0.71	0.74	0.65	0.72
1997	0.88	1.00	0.62	0.71	0.82	0.65	0.78
1998	0.82	0.96	0.89	0.66	0.55	0.41	0.74
1999	0.79	0.97	0.73	0.71	0.52	0.55	0.74
2000	0.75	1.02	0.57	0.80	0.41	0.61	0.76
2001	0.79	0.97	0.64	0.85	0.48	1.37	0.71
2002	0.74	0.93	0.59	0.74	0.51	0.41	0.81
2003	0.79	1	0.71	0.79	0.60	0.41	0.71
2004	0.75	0.95	0.65	0.75	0.60	0.29	0.74
2005	0.78	0.93	0.66	0.69	0.73	0.40	0.71
2006	0.77	0.94	0.68	0.67	0.65	0.27	0.77
2007	0.81	0.94	0.78	0.68	0.68	0.32	0.76
2008	0.78	0.93	0.75	0.64	0.65	0.27	0.69
2009	0.80	0.92	0.70	0.65	0.80	0.27	0.77
2010	0.77	0.90	0.61	0.66	0.79	0.59	0.76
2011	0.80	0.89	0.70	0.69	0.84	0.31	0.60
2012	0.79	0.89	0.73	0.65	0.77	0.34	0.68
2013	0.79	0.86	0.75	0.67	0.73	0.46	0.69
2014	0.76	0.85	0.68	0.66	0.72	0.41	0.7
2015	0.81	0.87	0.80	0.63	0.76	0.46	0.70
2016	0.78	0.82	0.74	0.67	0.78	0.51	0.62
2017	0.77	0.89	0.66	0.68	0.66	0.52	0.69
2018	0.97	0.84	-	-	-	-	-
2019	0.91	0.77	-	-	-	-	-
2020	0.93	0.78	-	-	-	-	-
2022	0.83	0.81	0.84	0.67	0.83	0.94	0.82

In the Table S9, "-" means no statistics are displayed.

Table S10. TME-based clinical trials.

ID	Title	Status	Interventions	Study Type
NCT02999178	Efficacy and Safety of Nintedanib in Patients with Progressive Fibrosing Interstitial Lung Disease (PF-ILD)	Complete	Drug: Nintedanib<br>Drug: Placebo	Interventional
NCT04046614	Feasibility and Safety of Nintedanib in Combination with Nivolumab in Pretreated Patients with Advanced or Metastatic NSCLC of Adenocarcinoma Histology	Complete	Drug: Nintedanib-nivolumab combination therapy	Interventional
NCT02989857	Study of AG-120 in Previously Treated Advanced Cholangiocarcinoma with IDH1 Mutations (ClarIDHy)	Complete	Drug: AG-120<br>Drug: Placebo	Interventional
NCT02368951	Phase I, Dose-escalation Trial of BAY1187982 in Subjects with Advanced Solid Tumors Known to Express Fibroblast Growth Factor Receptor 2 (FGFR2)	Terminated	Drug: BAY1187982	Interventional
NCT06626269	Identification of Innovative Biomarkers Related to the Immune System or Tumor Microenvironment to Promote the Efficacy of Immunotherapies	Not yet recruiting	Diagnostic test: Blood sample<br>Other: Tumor tissue	Interventional
NCT05955196	Test of CD47-SIRPα Inhibitors on the Immune Microenvironment Colon Cancer	Recruiting	Procedure: Blood and tumor	Observational
NCT06271421	NanoTherm In Adjuvant Therapy of Glioblastoma Multiforme	Recruiting	Device: NanoTherm therapy<br>Procedure: Glioma resection<br>Radiation: Radiotherapy according to Stupp protocol<br>Drug: Chemotherapy according to Stupp protocol	Interventional
NCT05816694	The Efficacy and Safety of Nanoparticle Albumin-bound (NAB)-Paclitaxel Plus Cisolation Versus CEP (Cisplatin, Epirubicin, Cyclophosphamide) in Induction Therapy for Thymoma: A Study for a Single-center Prospective Phase II Randomized Controlled Train	Not yet recruiting	Drug: NAB-Paclitaxel plus Cisplatin<br>Drug: Cisplatin plus Epirubicin plus Cyclophosphami de	Interventional

The contents of Table S10 are obtained from the ClinicalTrials.gov database (https://clinicaltrials.gov accessed on 23 November 2024).
